# A practice framework for the cooperative treatment of cancer between traditional health practitioners and radiation oncologists in KwaZulu-Natal province, South Africa

**DOI:** 10.4102/hsag.v26i0.1427

**Published:** 2021-02-25

**Authors:** Pauline B. Nkosi, Maureen N. Sibiya

**Affiliations:** 1 Department of Radiography, Faculty of Health Sciences, Durban University of Technology, Durban, South Africa; 2 Department of Nursing, Faculty of Health Sciences, Durban University of Technology, Durban, South Africa

**Keywords:** cooperative practice, cancer treatment, radiation oncologists, traditional health practitioners, framework analysis

## Abstract

**Background:**

Cooperative practice between traditional health practitioners (THPs) and radiation oncologists (ROs) is crucial for the continuity of care in the treatment of patients with cancer. However, scant information exists on how to co-ordinate cooperation between these health practitioners without interrupting the treatment of the patients.

**Aim:**

The study aimed to explore the practices of THPs and ROs in cancer treatment and ultimately derive a workable practice framework between these health practitioners in the KwaZulu-Natal (KZN) province.

**Setting:**

The study was conducted in selected districts, namely eThekwini, uThukela, Amajuba, uMkhanyakude, iLembe, uMzinyathi and uMgungundlovu, in KZN.

**Methods:**

A qualitative study by using a descriptive phenomenological approach was conducted to collect data from 28 THPs involved in the treatment of cancer and four ROs from public oncology hospitals. Focus groups and one-on-one semi-structured interviews by using open-ended questions were conducted to collect data from THPs and ROs, respectively. Framework analysis was used for data analysis to identify themes.

**Results:**

The study found that in KZN, THPs and ROs are working in parallel and that there are problems when patients seek cancer treatment from both health practitioners. Furthermore, the THPs and ROs work in an environment where there is no relationship, respect and trust, open communication and referral of patients by ROs to THPs. Both teams indicated that patients consult both traditional medicine (TM) and allopathic medicine (AM) by moving between the two health practitioners, resulting in interruptions in treatment. In addition, the study found that cooperation between THPs and ROs is understood as the provision of continuity care, where the parties work independently but share certain information of the patient on treatment, or as already being treated by each of them. The focus was on the type of relationship, enablers and common grounds for cooperation.

**Conclusion:**

The workable cooperative practice framework could be an inclusive health system where the parties work in parallel, with the patient being the main actor in the collaboration.

## Introduction

Cancer is a global concern because of its morbidity and mortality in the population. With conventional treatment, a patient diagnosed with cancer requires allopathic medicine (AM) and follow-ups for months, and frequently years, after the diagnosis of cancer (International Agency for Research on Cancer [IARC] 2018). The South African Constitution gives patients the right to access a health provider of their choice for healing purposes (Republic of South Africa 1996). In so doing, patients with cancer move between traditional health practitioners (THPs) and radiation oncologists (ROs), using both systems simultaneously or consecutively to search for diagnosis, healing or other services (Adams et al. 2009). Yet, these health practitioners do not communicate with each other. Consequently, the treatment is often disrupted and incomplete, thereby compromising the survival of patients (Merriam & Muhamad 2013; Pace et al. 2015).

Previous studies on cancer management showed that cancer can be controlled if the treatment services are accessible to all the inhabitants of a country, with cost-effective local treatment programmes being accessed by referral pathways (Sankaranarayanan et al. 2010). Such services should allow collaboration between the parties involved in cancer treatment and could build cancer treatment proficiency in the local communities (El Saghir et al. 2014; Kulendran et al. 2013). Moreover, collaborative practice will encourage referrals of cancer patients between the two teams (Nkosi & Sibiya 2018a), provide continuity of care (Bowles et al. 2008) and result in an expanded workforce, expanded access to care and opportunities for palliative care, exclusively in developing countries where there are constrained radiotherapy services (Trimble & Rajaraman 2017). Other studies concluded that the synergy between the treatments offered by THPs and ROs could improve the quality of healthcare in patients diagnosed with cancer (Fokunang et al. 2011; Van Rooyen et al. 2015).

However, challenges such as the quality of healthcare, differences regarding concepts of sciences and sources of knowledge, a lack of policy on collaboration, the lack of formal collaborative mechanisms and continuous wrestling with locally developed practices of THPs in cancer treatment (Nemutandani, Hendricks & Mulaudzi 2016) impinge on cooperative practice between THPs and Allopathic medicine practitioner (AMPs). The future of the health system in the effective treatment of patients with cancer is dependent on health practitioners changing fundamentally in their cooperative practice. Despite the challenges in cooperation, THPs are willing to cooperate in the treatment of diseases (Gqaleni et al. 2007; Krah, De Kruijf & Ragno 2018; Nkosi & Sibiya 2018b; Steyn & Muller 2000). Cooperation is needed between the THPs and ROs to refer patients with cancer properly, without interrupting the treatment of the patients. In addition, collaboration is needed to capacitate the radiotherapy services in the province because in KZN, there were seven ROs in public oncology hospitals, which provide cancer treatment compared with 15 000 active THPs (Gqaleni et al. 2007).

In 2002, the World Health Organization (WHO) devised a strategy for, amongst others, the integration of traditional medicine (TM) into the national health system to strengthen the health system of the country (WHO 2002). The literature indicates that cooperative practice can be described by looking at the key elements in the processes needed to work together. D’Amour et al. (2005:127) posit that these factors are determined by the type of collaboration and partnership, the degree of patient participation and are influenced by various factors arising from the external environment.

The exact definition of the cooperative practice required between THPs and ROs to ensure that patients with cancer complete their treatment has not been established by previous studies. Most studies on cooperation have been conducted in economically developed countries and focussed on explaining cooperation in general and partnerships, leaving a gap in determining the effectiveness of these in practices of service delivery (Sullivan & Skelcher 2002). In South Africa, previous studies have shown that THPs and AMPs in KZN have cooperated in the management of human immunodeficiency virus (HIV) and acquired immune deficiency syndrome (AIDS) and mental illnesses (Gqaleni et al. 2011; Mngqundaniso & Peltzer 2008) and that there were challenges to achieving effective cooperation. The aim of the study was to explore the practices of THPs and ROs in the treatment of patients with cancer to describe a viable cooperative practice framework between these health practitioners in the treatment of patients with cancer. Of the seven theoretical frameworks to study cooperative practice (Miller 1997), the Certified Nurse-Midwife, Physician and Client Collaborative Cycle was used to guide the study. Knowledge obtained from this study would be informative to future researchers wanting to develop cooperative practices that can be tailored to the agreement between the parties involved in the cooperative treatment.

## Methods

This article is part of qualitative interview data from a PhD study where data were collected between June and December 2015. A qualitative design by using a descriptive phenomenological approach to explore and describe the participants’ lived experiences (Pascal et al. 2011) of cancer treatment was employed. The philosophical underpinning of the study was the constructivism paradigm because the researchers believe that participants’ opinions and experiences of treating cancer patients would provide meaningful explanations of how the problem could be solved and an understanding of cooperative practice needed to solve the problem (Creswell 2009).

The study was conducted in the KwaZulu-Natal (KZN) province, South Africa’s second largest province in population size, which is organised into 11 districts (South Africa Info 2012) and has a population of 11.5 million (Statistics South Africa 2020). In 2007, there were 14 941 active THPs (Gqaleni et al. 2007) distributed across all 11 districts in KZN. The number of THPs in KZN at the time of the study was undocumented. Those who were interviewed were located at uThukela, Amajuba, uMkhanyakude, iLembe, uMzinyathi districts and uMgungundlovu districts. There were seven ROs in the public oncology hospitals in KZN and of those, four participated in the study. The description of THPs and ROs, their selection and how the researchers collected data from them were discussed in a previous study conducted in KZN (Nkosi & Sibiya 2018a).

Data were collected by using focus groups and face-to-face one-on-one semi-structured interviews on THPs and ROs, respectively. They were collected between September 2015 and December 2015. The interviews were conducted at the research setting. All THPs were interviewed in isiZulu by the researcher because all participants spoke isiZulu. No interpreter was needed as the researchers’ mother tongue is similar to the participants’. On the other hand, the ROs were interviewed in English because this was their first language and the researchers did not require an interpreter as they are conversant in this language.

The interviews for both participants comprised two sections, namely demographic data and information on their experiences in cancer treatment. In the demographic data section, demographic information of participants was captured. In the second section, the researchers used open-ended questions such as ‘What is your experience in treating patients with cancer who consult with both health practitioners consecutively or simultaneously?’ and ‘What would constitute a workable practice framework in cancer treatment between the groups of health practitioners involved in cancer treatment?’ The researchers probed the participants during the interview by using Miller’s theoretical framework, namely the Certified Nurse-Midwife, Physician and Client Collaborative Cycle (D’Amour et al. 2005). These included external conditions, individual characteristics, organisational dynamics, trusting attitudes and viewpoints of practice. Collected data from THPs were translated back to English before data analysis.

Data were first transcribed verbatim in full-text and then analysed in a step-by-step approach by using the framework analysis. A framework analysis is used to manage the massive data in health science research and ensures systematic qualitative data analysis (Smith & Firth 2011) in the fields of nursing, psychology and sociology. According to Caroll, Booth and Cooper (2011), this involves primary identification of *a priori* themes against which to chart the data and represent the platform whereupon the findings may be brought together and organised. This enables the researcher to explore data in-depth whilst simultaneously maintaining an effective and transparent audit trail, enhancing the rigour of the analytical processes. According to Caroll et al. (2011), in the interpretation stage, a framework synthesis is utilised to find the relationship between the sub-themes that emerged in the data analysis. Ensuring that data analysis is explicitly described enhances the credibility of the findings. The researchers integrated all major categories to form a larger practice framework.

### Trustworthiness

To safeguard the trustworthiness in the study, the researchers ensured that from data collection to data analysis, the research fulfilled four criteria, namely credibility, dependability, confirmability and transferability (Polit & Beck 2013). To ensure credibility in this study, the data triangulation method was used. In data triangulation, many sources of information are used to increase validity in the study. In addressing dependability, the research design of this study might be viewed as a ‘prototype model’ to enable researchers to develop a thorough understanding of the methods and their effectiveness. To achieve confirmability, the researchers ensured that the study findings were the results of the lived experiences that emerged from the THPs and ROs who treat cancer by conducting in-depth interviews and generating thick descriptions. All responses were recorded, categorised and compared with items in the refined coding system. Excerpts and direct quotes from the data were used to support the themes that emerged from the data. The supervisors were invited to review the data scripts. The researchers and the supervisors concurred on the identified categories and themes. For transferability, the researcher established the context of the study and gave a detailed description of the phenomenon by interviewing 28 THPs and 4 ROs with various experiences to allow comparisons to be made.

### Ethical considerations

The Institutional Research Committee of the Durban University of Technology granted ethical approval for conducting the study (Ethics clearance reference number: REC 1/15). Permission to conduct the study was requested from the District Manager and KZN Department of Health. Gatekeeper permission was granted by the Chief Executive Officers at the public hospitals in which the ROs were placed. All participants were given letters of information and signed consent before participating in the study. Their participation was voluntary and they were told that they could withdraw at any time if they so wish, without any penalty. The researcher audio-recorded the interviews with the permission of the participants. Anonymity and confidentiality were maintained throughout the research project.

## Results

**FIGURE 1:**
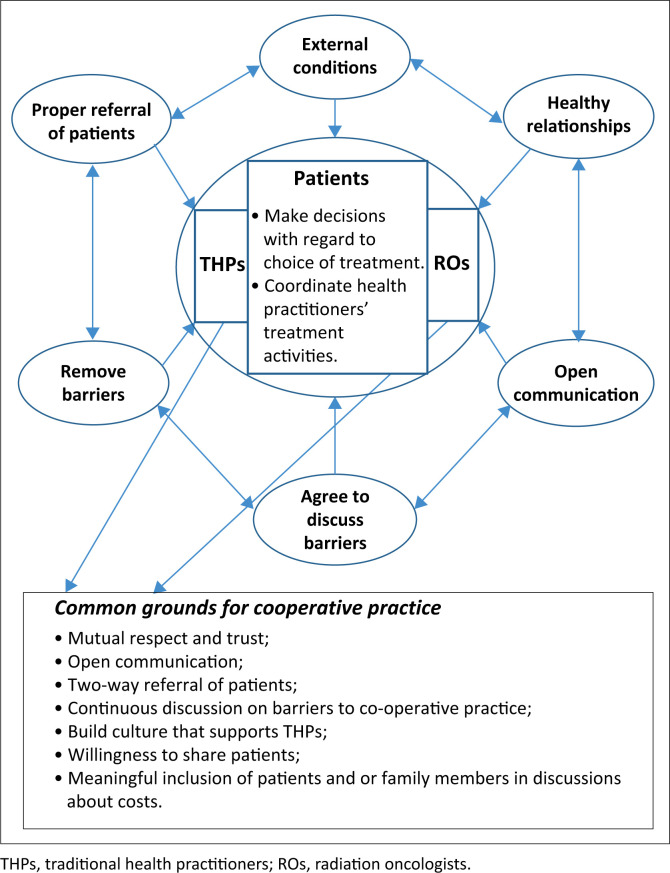
The framework for cooperative practice between traditional health practitioners and radiation oncologists in the treatment of cancer patients in KwaZulu-Natal.

The analysis is based on the cooperative practice between THPs and ROs and the framework for cooperative practice. Cooperation between THPs and ROs in the treatment of cancer is understood as the provision of continuity care where the parties work independently but share certain information of the patient on treatment, or who has already been treated by each of them. The approaches used were the parallel working relationship whereby the parties work as a team with the patient at the centre, responsible for the coordination of treatment activities between the healthcare practitioners. In addition, the practice framework describes the enablers and common grounds for cooperation and the referral of patients between them (Figure 1).

### Strategies for effective collaboration between THPs and ROs in the treatment of cancer patients in KZN

The practice framework developed describes the type of relationship, enablers and common grounds to ensure that the health practitioners collaborate in the cancer treatment.

#### Type of relationship

Almost all THP and RO participants were willing to cooperate with each other, but they did not know the appropriate type of collaboration. The following excerpts confirm this:

‘We need to work together; however, the government should first have evidenced-based role of our treatment for cancer patients, then use the law for us to work together.’ (THP 5, male, traditional herbalist)

‘With regard to co-operative practice, we do want to work with them as mandated by the government; however, it is difficult …’ (RO 2, female, 38 years)

All the participants indicated that the patient was the main actor in the cooperative practice. The following extracts illustrate this:

‘Involve the patient in decision-making, and this should involve the patient and the two health practitioners. We need to explain to the patient how the patient should be treated. Once the diagnosis of the patient is confirmed, the patient should be told about the role of THPs and ROs in the treatment of cancer, then the patient should make an informed decision with regard to a health practitioner of their choice, then sign documents to that effect.’ (THP 15, male, diviner)

‘… However, some patients indicated that they have been to THP, but we don’t know what form of medication or treatment they have received as well as the duration of the treatment and the role of treatment in cancer. They need to provide us this information.’ (RO 4, male, 46 years)

#### Enablers for cooperative practice

The ROs viewed enablers as the external conditions that mandate cooperative practice and, therefore, are of the opinion that it is the government’s responsibility to facilitate cooperative practice. The following excerpt confirmed this:

‘The government in 1994 gave THPs permission to work with us, however, we don’t know the limits of that permission … we would like to know what their vision is about that and what THPs’ perception in treating cancer … We don’t know how this treatment is applied; there is no guideline or recommendations we can study or learn from research about this.’ (RO 1, male, 45 years)

In working together, the participants believed that the following issues need to be considered and addressed.

### Healthy relationship between the parties in the cooperative partnership

The interviewees believed that there should be a relationship amongst them to facilitate referral of patients. The following extracts support this:

‘… we don’t know who to refer to at the hospitals. I think they also don’t know us. We have realised that the referral is a one-way system from THPs only and not the hospitals.’ (THP 2, male, traditional herbalist)

‘I have not seen too many patients that were referred by THPs. Some patients have told us that they have had TM treatment before coming to us but we don’t know any details of this treatment like how long it was given.’ (RO 4, male, 46 years)

### Open communication between the health practitioners

Most THPs interviewed were of the opinion that there should be open communication when they work together. This is indicated by the following quote:

‘The patient should be counselled and told that we are working together. In that way they will be able to tell them about their visit to us.’ (THP 3, male, traditional herbalist)

### Agree to discuss and remove barriers to cooperative practice

Both THP and RO interviewees stated that for effective cooperative practice, they need to discuss and remove barriers emanating from the perceptions of each team of health practitioner about the other in the treatment of cancer patients. Most THPs interviewed highlighted many other issues to be involved in the discussion to facilitate co-operative practice. The following statements support this:

‘We need to get together with ROs to discuss that some patients will not be cured because of their ancestors.’ (THP 22, female diviner)

### Common grounds for cooperative practice

‘We need to get together with ROs to discuss how we can treat cancer patients successfully. The cancer patients should be counselled and told that we are working together. In that way they will be able to tell them about their visit to us. Also, they need to be workshopped on how they will be referred between the two health practitioners. We need to educate each other about cancer and that some cancers cannot be cured because of the ancestors’ spirit. Having this shared knowledge, we can approach cancer successfully.’ (THP 23, female traditional herbalist)

‘… to achieve this, we need to work together with ROs because amongst them there are those who cannot cure the disease and the same applies to us THPs and find common grounds. We need to discuss this together.’ (THP 7, male traditional herbalist)

‘Doctors don’t want work with us because they perceive us as uneducated people who have demons, yet we do believe in the Bible.’ (THP 14, female diviner)

‘We need to interact; however, the government should first have evidence-based role of our treatment for cancer patients, then use the law for us to work together.’ (THP 25, female diviner)

The ROs viewed the following factors as the common ground for cooperation, as illustrated by the given excerpts:

‘If the THPs want to collaborate with us we don’t have a problem, we can talk, but they must understand that evidence-based practice should be the priority in our discussions. There is no proof that TM or their practices can be used in cancer patients. What we practise is evidence-based … I cannot recommend this for now because of there is no evidence that it can be used in cancer treatment.’ (RO 1, male, 45 years)

‘They need to tell us how they treat cancer and tell us their role. We can then discuss the way forward together.’ (RO 2, female, 38 years)

### Proper referral of patients between the traditional health practitioners and radiation oncologists

‘We need to work together with the doctors so that we can conquer cancer. In so doing we need to discuss how to refer patients, share responsibility and share information as to whom amongst us are the cancer treatment specialists and the type of cancers we can treat successfully.’ (THP 22, female, both traditional herbalist and deviner)

‘I do not have any experience of treating a patient who was being treated by a THP and they have not referred any patient to me … The problem is that after being diagnosed with the disease they disappear and return when the disease has advanced. This makes it difficult for us to manage the disease. When working together, the THPs should refer patients early to avoid delaying the diagnosis and treatment which results in patients presenting in advanced stage.’ (RO 3, male, 46 years)

## Discussion

This study analysed the cooperative practice between THPs and ROs in cancer treatment to develop a framework for effective collaboration. The study found that in KZN, THPs and ROs are working in parallel and that there are problems when patients seek treatment from both health practitioners. According to Nkosi and Sibiya (2018b), THPs and ROs work in an environment where there is no relationship, respect and trust, open communication and referral of patients by ROs. Both teams indicated that patients consume both TM and AM by moving between the health practitioners, thereby resulting in interruption of treatment, delay in diagnosis and patients presenting with advanced disease. Trimble and Rajaraman (2017) illustrated this by stating that the delays in cancer diagnosis occur with both health practitioners and, therefore, both should consider the significance of prompt diagnosis.

The researcher noted that the health practitioners recognised that patients have a right to consult a health practitioner of their choice, as the South African government mandated them to cooperate and they need to respect the culture of patients to provide continuity of care. Findings in this study show that they are willing to collaborate, but they require the government to facilitate this collaboration. This demonstrates a positive attitude towards incorporating TM into the national health system. This finding resonates with the findings by Merriam and Muhammad (2012) where the authors suggested that the government should provide regulations and guidelines for THPs to work together with AM practitioners.

A change is needed in the relationship where the parties cooperate with each other, interact and work together in harmony, where there should be mutual trust and respect to effect the referral of patients between the health practitioners. There should be open communication on matters related to treatment, and treatment activities should be coordinated to ensure continuity of care for patients. The parties should agree to discuss and remove barriers to effective cooperation. Furthermore, there should be a two-way referral of patients between the health practitioners to refer a patient when the treatment is unsuccessful, hence reducing delays in the referral of patients between the health practitioners.

This study also shows that the THP and RO participants understood cooperative practice differently in a collaboration. They described it in terms of interaction and the referral of cancer patients between them. The researcher noted that the participants used the terms ‘work together’ and ‘work with’ interchangeably, yet their understanding of these terms have different meanings for different participants. According to Sherman et al. (2017), in a cooperative practice, authors wrestle with terminology to describe collaboration. Despite this, THPs in the eThekwini Metropolitan Health District of KZN are enthusiastic to share their knowledge with AM practitioners (Gandugade, Nloot & Naidoo 2017).

Strategies that are used for co-operative practice between THPs and ROs in the treatment of cancer aimed to ensure effective cooperation between these health practitioners in the KZN province. They include describing the type of collaboration and the environment in which it should occur. The framework developed explores the environment for effective cooperation and describes external conditions, enablers and common grounds for cooperation. The five conceptual categories for understanding co-operative practice that emerged from the data analysis process were external conditions, healthy relationships, open communication, the agreement to discuss and remove barriers to collaboration and the proper referral of patients.

The researcher noted that for effective cooperative practice, the parties should know each other, interact, refer patients to each other and work together. Both the THP and RO participants highlighted that working together involves both parties, with the patient playing a major role in the relationship. According to Nkosi and Sibiya (2018b), the patient makes decision about the type of health practitioner to provide treatment, communicates between the healthcare practitioners and coordinates their activities. However, the ROs did not indicate a need to refer their patients to the THPs although they want to provide continuity of care to patients. To support this finding, Habtom (2018) stated that a country’s healthcare system that recognises TM but is not yet fully integrated into all aspects of healthcare is an inclusive one. This indicates the type of collaboration that they are willing to adopt.

The framework developed provides strategies and ideas that would assist healthcare practitioners to adopt the elements of the collaboration and cooperative practice that they deem would be beneficial. It, therefore, identifies the elements of collaboration, enablers for successful collaborative teamwork and outlines several action items that policymakers can apply within their local health systems. The framework could be adapted and utilised by healthcare providers to improve on referrals by the ROs, who were found to be lacking in this regard.

## Conclusion

The study analysed the practice of THPs and ROs in the treatment of cancer patients and ultimately developed a framework for cooperative practice between these healthcare practitioners in the treatment of cancer patients in KZN, South Africa. Although THPs and ROs were willing to cooperate for effective practice, the process of integrating TM into the national health system is time-consuming because of discussions on the external conditions that support cooperative practice, building a healthy relationship between the two teams, open communication, referral of patients between the health practitioners and the removal of barriers to cooperative practice. Parties contemplating to initiate collaboration should first know each other, discuss barriers to cooperative practice and find common grounds for cooperative practice. The developed practice framework can be used to improve the relationship between the health practitioners, enable the establishment of a proper referral system, increase the capacity of cancer control and strengthen the national health system in South Africa. Furthermore, effective cooperation is time-consuming and requires commitment from the parties involved.
